# Major Histocompatibility Complex and Hematopoietic Stem Cell Transplantation: Beyond the Classical HLA Polymorphism

**DOI:** 10.3390/ijms19020621

**Published:** 2018-02-22

**Authors:** Alice Bertaina, Marco Andreani

**Affiliations:** 1 Department of Pediatric Hematology and Oncology, IRCCS, Ospedale Bambino Gesu’, 00165 Rome, Italy; 2 Division of Stem Cell Transplantation and Regenerative Medicine, Department of Pediatrics, School of Medicine, Stanford University, Stanford, CA 94305, USA; 3 Laboratory of Immunogenetics and Transplant Biology, IME Foundation, Policlinic of the University of Tor Vergata, 00133 Rome, Italy; m.andreani@fondazioneime.it

**Keywords:** human leukocyte antigens (HLA), major histocompatibility complex (MHC), hematopoietic stem cell transplantation (HSCT), anti-HLA antibodies, natural killer (NK) cells

## Abstract

Allogeneic hematopoietic stem cell transplantation (HSCT) represents a curative treatment for many patients with hematological malignant or non-malignant disorders. Evaluation of potential donors for HSCT includes a rigorous assessment of the human leukocyte antigens (HLA) match status of family members, and the identification of suitable unrelated donors. Genes encoding transplantation antigens are placed both within and outside the major histocompatibility complex (MHC). The human MHC is located on the short arm of chromosome 6 and contains a series of genes encoding two distinct types of highly polymorphic cell surface glycoproteins. Donors for HSCT are routinely selected based on the level of matching for HLA-A, -B, -C, -DRB1, and -DQB1 loci. However, disease relapse, graft-versus-host-disease, and infection remain significant risk factors of morbidity and mortality. In the same breath, in high-risk patients, graft-versus-leukemia effects inherent in HLA mismatching play a substantial immunological role to limit the recurrence of post-transplant disease. The definition of a suitable donor is ever changing, shaped not only by current typing technology, but also by the specific transplant procedure. Indeed, a more complete understanding of permissible HLA mismatches and the role of Killer Immunoglobulin-like receptors’ genes increases the availability of HLA-haploidentical and unrelated donors.

## 1. The Human Major Histocompatibility Complex (MHC)

The human major histocompatibility complex (MHC), known as human leukocyte antigen (HLA) complex, constitutes a specific group of molecules expressed on the cell surface that is crucial for the recognition of non-self molecules by the acquired immune system. Therefore, HLA molecules’ essential function is to bind and display antigens derived from pathogens on the cell surface, in order to present them to the appropriate T lymphocytes. From a genetic point of view, HLA is a cluster of more than 200 genes [1] located on chromosome 6 (6p21.3) which extends for about 4 Mb, distinguished in three major groups, HLA class I, HLA class II, and HLA class III. The HLA class III region’s genes encode proteins which differ in both structure and function to the class I and class II loci, including complement components, several inflammatory cytokines, such as tumor necrosis factor α (TNFα) or the heat shock proteins. The class I region contains the class I classical genes, such as HLA-A, -B, and -C besides the class I non-classical genes including HLA-E, -F, -G, -J, -X, MICA, MICB, MICC, MICD, and MICE. HLA class I antigens are composed of three α chain domains: α1, α2, and α3, besides a non-MHC molecule β2 microglobulin, encoded on human chromosome 15. The α3 domain is transmembrane, anchoring the molecules to the cell membrane, while the central region of both the α1 and α2 domains form the floor of the peptide-binding groove, which, based on genetically encoded and expressed sequence of amino acids, determines which peptide residues bind and the level of its affinity. The HLA Class II genes consist of two chains, α and β, both formed in two domains: α1, α2, β1, and β2. The transmembrane domain, α2 and β2, respectively, anchor the HLA class II molecule to the cell membrane, while the peptide-binding groove is formed by the heterodimer α1 and β1 [2]. The best characterized of these genes, HLA-DPB1, -DQA1, -DQB1, and -DRB1, encode proteins that display processed antigenic peptides for recognition by T helper cells. The binding of peptides with the HLA molecules is based on the interaction that occurs between the amino acid residues of both the MHC groove and of the peptides. The groove of the HLA molecules contains different pockets, defined as the unit having an affinity for a specific peptide side chain. The pockets may show differences in size, shape, and function [3]. Some of them have affinity for a group of side chains, while others have an affinity only for one side chain. These last are known as anchor residues specific for anchor positions, which, based on identity and spacing, constitute the peptide motif of an HLA specificity [4,5,6,7]. Therefore, the HLA alleles differ from each other by substitutions in amino acid residues, contributing to the specific structure of the peptide-binding pockets and as a consequence, to differences in immune responses among individuals. One of the most interesting characteristics of the HLA system is the extensive level of variation of the loci number, and the number of alleles at those loci. The IPD-IMGT/HLA website database 3.26.0, released on October 2016, reported that the extensive polymorphism of the MHC molecules was constituted by 15.420 different HLA-A, -B, -C, -DRB1, -DRB3/4/5, -DQA1, -DQB1, -DPA1, -DPB1 alleles [8]. Interestingly, based on the genetic phenomenon of denominated linkage disequilibrium (LD), despite their frequencies, many alleles of the different loci are present in individuals more frequently than expected by random distribution, inherited in blocks, known as haplotypes, varying among the ethnic groups [9].

## 2. The Role of Human Major Histocompatibility Complex (MHC) in Allogeneic Hematopoietic Stem Cell Transplant

Allogeneic hematopoietic stem cell transplantation (HSCT) represents a successful treatment option for either malignant and non-malignant hematologic or immune disorders. Among many factors that influence the clinical successful of HSCT, polymorphism of the classical HLA genes represents one of the major barriers, leading physicians to the selection of an HLA potential compatible donor, in order to reduce the risk of graft failure, graft-versus-host disease (GvHD) and mortality and providing a better probability of disease-free survival [9,10]. Unfortunately, due to the great diversity of HLA alleles and haplotypes, the possibility of identifying an unrelated donor matched at allelic resolution remains a difficult challenge for most patients [11,12]. A HLA genotypically identical sibling donor is, therefore, the best choice, although only 25–30% of the patients have this option available. Over the past three decades, the extent of allelic diversity at HLA loci has been well characterized using high-resolution HLA-DNA typing [13], particularly concerning the volunteer donors enrolled in the international registries, that, up to date, account for more than 27 million. HLA typing by next-generation sequencing (NGS) methods is likely to improve donor–recipient matching by providing full sequence information on all HLA loci in a shorter period of time compared to other methods. The routine use of NGS for HLA typing will definitely improve the identification of well-matched unrelated donors and will further develop the HSCT programs. However, keeping in mind that the definition of HLA matching is relative to the level of resolution used for the search in the different international donor registries and to the number of loci analyzed which characterize the extremely great diversity of HLA alleles and haplotypes, for many patients it will still be difficult to find a fully HLA-matched donor. Helpful for solving ambiguities, taking into account any worldwide HLA allele distribution, are the two available catalogues of common and well-documented (CWD) HLA alleles; the first established by the American Society for Histocompatibility and Immunogenetics (ASHI) [14], the second by the Population Genetics Working Group of the European Federation for Immunogenetics (EFI) [15]. Both the two different catalogues report a similar number of CWD alleles (EFI: 1048 vs. ASHI: 1031) but the one produced by EFI shows a lower number of common alleles (236 vs. 377) and a higher number of well-documented (812 vs. 654) alleles than the one from ASHI, possibly reflecting differences in sample numbers and sizes. Interesting to note is that about 50% of the CWD alleles reported in the EFI catalogue are different from the ASHI one, underlining the distinct features of the ethnicity investigated. Moreover, the nomenclature update, (Nomenclature for factors of the HLA system, 2010) [16] provides helpful aid for reporting of identical peptide binding domains or of identical nucleotide sequences in specific allele strings. Therefore, the upper case ‘P’ will indicate HLA alleles showing an identical nucleotide sequence encoding for the same protein sequence of the peptide binding domains, while the upper case ‘G’ will characterize HLA alleles with identical nucleotide sequences for the exons encoding the peptide binding domains (for HLA class I—exon 2 and 3; for HLA class II—exon 2). In both cases the allele designation will be named with the lowest numbered allele in the group. It is a well-accepted concept that single HLA mismatches for any single locus have a clinical impact on the HSCT outcome. In fact, large international studies have shown that single mismatched incompatibilities in respective HLA loci between donor and patient produce individualized differences in clinical results [17]. Nevertheless, contradictory information regarding the benefits of choosing a donor with an allelic or an antigenic mismatch has been reported in literature. In a recent paper, Morishima et al., analyzing 7898 Japanese pairs transplanted from a matched unrelated donor (MUD) characterized by a complete HLA allele typing data, showed a significant relative risk in HLA allele mismatch, compared with match at HLA-A, -B, -C, and -DPB1 loci, for grade III-IV GvHD, and HLA-C for chronic GvHD. A significantly increased risk for mortality and aGvHD was associated to the presence of double mismatch for HLA-DRB1 and HLA-DQB1. [18]. On the other hand, Lee et al., excluding the impact of C locus, showed that there were no significant differences on survival based on allelic or antigenic mismatches between donor and patients [9]. Pidala et al., analyzing a survey of 8003 patients, observed that a single mismatch at HLA-B had a greater risk of grade III-IV acute GvHD, chronic GvHD and a lower risk of relapse compared to single mismatch at HLA-C, with no further statistically significant difference due to the presence of other mismatched loci [19]. Moreover, considering the potential risk of antigen vs. allele mismatching, they observed an increased risk of grade II–IV aGvHD in pairs with a B antigen mismatch, compared to those with a B allelic mismatch. A study from the Center for International Blood and Marrow Transplant Research (CIBMTR) evaluating HSCT outcome, employing mainly bone marrow (BM) as a stem cell source in myeloablative conditioning regimen, suggested that a single HLA-A and HLA-DRB1 mismatch appeared to be detrimental in comparison with a single mismatch at HLA-B or HLA-C [9]. Furthermore, a study investigating the impact of HLA mismatches in peripheral blood stem cell (PBSC) transplant recipients [20] showed a higher risk of mortality in those receiving an allograft from a donor presenting one antigen mismatch, either in the HLA-C or HLA-B loci. In a recent review, Tiercy defined the negative impact of a single HLA-C mismatches in unrelated HSCT, with a reported mortality of 21–43%. In the vast majority of the transplant centers, unrelated donor search algorithms now also take into account HLA-C compatibility, in view also of the HLA–B–C associations in specific haplotypes. It has been shown that the immunogenicity of HLA-C mismatches in patient-donor pairs might be due to differences at class I molecule residues. Among these, residue 116 in the F pocket of the peptide binding groove has been identified as having a high frequency of mismatches that are responsible for adverse clinical transplant outcome, such as risk of acute GvHD and mortality, in comparison to HLA-C-matched donor-recipient pairs [21]. In order to determine the impact of each HLA allele mismatch combination in inducing acute GvHD, Kavase et al. analyzed a total of 5210 donor/recipient pairs who underwent HSCT through Japan Marrow Donor Program, typing retrospectively all the HLA-A, -B, -C, -DRB1, -DQB1, and -DPB1 alleles. They were able to determine 15 different high-risk HLA allele mismatch combinations and 1 HLA-DRB1-DQB1 linked mismatch combinations correlated with the occurrence of severe acute GvHD. Moreover, they identified 6 different amino acid substitutions, located at positions 9, 77, 80, 99, 116, and 156 of the HLA class I molecule, that are responsible for aGvHD. Although controversial, they speculated that severe acute GvHD might be associated between ligand matching of the NK-cell receptor (KIR2DL) and positions 77 and 80 in HLA-C [22]. In a different study, Fernandez-Vina et al. showed similar HSCT outcome in patients typed as C*03:03/C*03:04 mismatched compared to a group of 8/8 HLA matched. Therefore, they suggested that mismatches in this specific HLA allele combination are better tolerated than other HLA mismatches, where higher risks of acute GvHD and lower survival are readily detectable [23]. Lazaryan et al. recently investigated the impact of HLA mismatches of the HLA grouping in supertypes, in consideration of the similarities present both in the predicted structure and function of the epitope binding grooves of HLA molecules. They categorized single mismatched alleles into six HLA-A (A01, A01A03, A01A24, A02, A03, A24), six HLA-B (B07, B08, B27, B44, B58, B62), two HLA-C (C1, C2), and five HLA-DRB1 (DR1, DR3, DR4, DR5, DR9) supertypes. They showed that supertype B07, B44 mismatch was associated with a higher incidence of both grade II–IV and III–IV acute GvHD, while no significant associations were identified between supertype-matched versus supertype-mismatched groups at other HLA loci [24]. Another issue to consider for successful HSCT is the expression of HLA molecules. It is well known that acquired immunodeficiency syndrome or susceptibility to Crohn’s disease are influenced by the expression level of the HLA-C on the cell surface [25]. Petersdorf et al. have recently shown, as impact on aGvHD after HSCT, that the presence of HLA-C mismatches involving alleles that have, on average, higher expression levels was more poorly tolerated than mismatches involving alleles that have, on average, lower expression levels. The effects of different expression levels at other HLA loci should be more thoroughly analyzed in the future, since it might increase the number of acceptable donors, especially for patients with hematological malignancies [26]. Recent analyses have shown how, according to T-cell epitope, donor-recipient combinations of HLA-DPB1 may be considered permissive or non-permissive, in particular for the risk of severe aGvHD and mortality [27,28]. Pidala et al., in a survey of 8003 patients confirmed the adverse impact of non-permissive DPB1 mismatch on either transplant-related or overall mortality, although the increased risk of aGVHD grade II–IV was associated with both permissive and non-permissive HLA-DPB1 allele mismatch, compared to matched cases [29]. Finally, as reported by Chuna et al. in a multivariate analysis relative to a survey of 565 patients who received a transplant from a single cord blood unit-unit, one or two HLA mismatches in the GVH or HVG direction were not associated with non-relapse related mortality and survival, compared with a 5/6 HLA matched population [30].

## 3. Natural Killer (NK) Cells and KIR/KIR Ligand Polymorphism

Natural killer (NK) cells are a lymphocyte population of innate immune cells capable of providing rapid responses against tumors and viral infections without prior sensitization. Moreover, they play a pivotal role in immunosuppression and tolerance towards fetus allograft [31,32]. Therefore, a substantial body of evidence has recently emerged delineating the role of NK cells in the immunosurveillance of leukemia. The role for NK cells in this setting is a direct consequence of their inherent biology. As a matter of fact, NK cells are a hallmark component of the innate immune system; their functions are regulated by a variety of germ-line-encoded activating and inhibitory cell surface receptors and can be modulated by cytokines/chemokines, PAMPs, and education mechanisms [33]. The first source of inhibitory signals comes from receptors that recognize self-HLA class I alleles. In humans, there are two main classes of inhibitory receptors: the killer immunoglobulin-like receptors (KIR) family specific for groups of classical HLA-A, -B, and -C allotypes and the heterodimer CD94/NKG2A. Recognizing non-classical HLA-E molecules that express peptides derived from leader sequences of different HLA class I molecules, CD94/NKG2A controls the overall expression of HLA class I on potential targets [34]. KIR are members of the immunoglobulin (Ig)-superfamily and their classification is based on the number of extracellular domains and the length of the cytoplasmic tail. The presence of immunoreceptor tyrosine-based inhibitory motifs (ITIMs) characterize the long cytoplasmic tails in Inhibitory KIRs (iKIRs). Upon KIR/KIR-ligand (KIR-L) recognition, ITIMIs become phosphorylated, and recruit tyrosine phosphates turning off NK cell responses. Conversely, activating KIRs (aKIRs) lack ITIMs and are connotated by a positively charged amino acid residue in the transmembrane region that allows for interaction with DAP-12, an adaptor signaling molecule with an immunoreceptor tyrosine-based activating motif (ITAM) [31,35,36]. Notably, the NK cell receptor repertoire enables NK cells to kill targets through altered expression of HLA class I and of upregulated stress-inducible antigens which prevents NK autoreactivity against self-targets [35].

NK cells are the first post-HSCT cellular population, reconstituting antiviral and antitumor activity [37]. In this setting, donor NK cell inhibitory receptors mismatched for cognate HLA-Class I ligands play a key role in the graft versus leukemia (GVL) effect [38]. Remarkably, these cells may be uniquely poised to enhance GVL without eliciting GvHD because healthy non-hematopoietic tissues lack activating receptor ligands present on tumor cells [37]. Over 20 years ago, Moretta et al. described the concept of NK cells’ alloreactivity, showing that defined NK cell subsets were able to kill in vitro allogeneic lymphoblasts [39]. Ruggeri et al. first reported the positive impact of KIR ligand mismatched donor NK cell alloreactivity after T cell-depleted HLA haploidentical HSCT (haplo HSCT) resulting in a lower risk of relapse and a better overall survival in adult with acute myeloid leukemia (AML) [40]. Of note, only patients receiving a transplant from a donor who showed NK cell alloreactivity against recipient cells, displayed an efficient graft-versus-leukemia (GVL) effect. This happens, for example, in the presence of a KIR-HLA-I (KIR-L) mismatch in the donor-versus-recipient direction. Thus, in donor/patient pairs with KIR-HLA-I mismatch, the event-free survival (EFS) rate was 60%, while in the absence of such mismatch, it was less than 5% [41]. However, the contribution of NK cell alloreactivity on HSCT outcome is still controversial due to different evaluation criteria, the nature of KIR/KIR ligand genetic combinations studied, and NK cell repertoire size [42,43]. Interestingly, Clausen et al. observed an opposite effect of missing KIR ligands in patients transplanted with bone marrow when compared to peripheral blood stem cells as graft source, in recipients homozygous for HLA-C group 2 ligands for inhibitory killer Ig-like receptors [44]. Recently, the same authors suggested that the use of anti-thymocyte globulin (ATG) may provide a survival benefit in recipients with at least one C1 group KIR-L, by reducing transplant-related mortality (TRM) without significantly increasing the relapse risk [45]. Given the central role of NK cell alloreactivity in preventing leukemia relapse, in the setting of haplo-HSCT it is crucial to determine in different potential donors if alloreactive NK cells are present and the size of such alloreactive populations. Nevertheless, it is fundamental to evaluate the post-HSCT generation of donor-derived alloreactive NK cells in the recipient [46,47]. The use of appropriate combinations of anti-KIR monoclonal antibodies (mAbs) in multicolor cytofluorimetric analysis allows the identification and the definition of the of the alloreactive NK subset size. Notably, since the presence of some activating KIRs is of positive prognostic value contributing to the lysis of leukemia blasts, the recent availability of mAbs discriminating between activating and inhibitory KIRs permit a more precise definition of the size of the efficient, alloreactive NK cell population [47,48,49]. More recently, in addition to the flow analysis of the alloreactive NK cell populations, other selection criteria have been added (Table 1). Among these, the presence of a KIR B haplotype has been shown to be associated with a relevant improvement of the survival in both adult AML [50] and pediatric acute lymphoblastic leukemia (ALL) [51]. In 2014, Overmann L. and colleagues analyzed the influence of donor KIR gene haplotypes on the outcome of pediatric patients with ALL given a T-cell-depleted peripheral blood stem cells haplo-HSCT. In this paper, the KIR gene haplotype was evaluated in 85 donors, while the KIR B content score was determined only in the 63 KIR haplotype B donors. Children transplanted from a KIR haplotype B donor had a significantly better EFS in comparison with those transplanted from a KIR haplotype A donor (50.6% vs. 29.5%, respectively; *p* = 0.033). Moreover, a high donor KIR B-content score was associated with a significantly reduced risk for relapse (Log-rank test for trend, *p* = 0.026). Overall, these data suggest that whenever possible, for haplo-HSCT in children with ALL a KIR haplotype B donor with a high KIR B-content score should be selected [51].

Thanks to the recent exponential application of haploidentical donors in both adult and pediatric age groups, the impact of NK cells on allogeneic HSCT is becoming even more important. Indeed, suitable HLA-compatible donors, whether siblings or matched unrelated, can be identified for approximately 60–70% of patients in need of HSCT. For the remaining population, either with high-risk leukemia or genetic non-malignant disorders, the prognosis historically has inevitably been very poor. At the end of 90 s, a major achievement for overcoming this problem was the observation that, for such patients, a family member identical for one HLA haplotype and fully mismatched for the other (i.e., haploidentical), may be immediately available as a stem cell donor. This is true especially for pediatric patients, occurring with 100% of parents and 50% of siblings. Critical for the success of the haplo-HSCT was the demonstration that an efficient T-cell depletion of the graft associated to high-intensity immunosuppressive/myeloablative conditioning and to the infusion of a ‘megadose’ of purified CD34^+^ resulted in high rates of engraftment and low incidence of mild acute GvHD (no grade II–IV), even in the absence of post-transplant immunosuppression [52]. Until 2010, haplo-HSCT has been performed mainly by using positively selected CD34^+^ cells. A delayed immune recovery and a subsequent increased risk of TRM compared with HLA-matched unmanipulated allografts were unsolved obstacles to the success of the procedure [53]. To overcome these hurdles, a novel method of ex vivo T- and B-cell depletion based on the physical elimination of αβ^+^ T cells through labeling with biotinylated anti-T-cell receptor-αβ (anti-TCR-αβ) and anti-CD19 antibodies, followed by incubation with anti-biotin antibodies conjugated to paramagnetic microbeads (CliniMACS; MiltenyiBiotec, BergischGladbach, Germany) was recently implemented in selected centers [54,55,56]. Remarkably, thanks to this novel graft manipulation approach, other mononuclear cell types are infused into the patients. Of major interest are γδT cells, a lymphocyte subset able of exerting GvL activity since, similar to NK cells, express the DNAM-1 and NKG2D activating receptors specific for ligands expressed on tumor cells. In addition, the Vδ2 population recognizes non-peptide phosphoantigens expressed by leukemia cells, whereas Vδ1 cells expand in response to CMV reactivation. The presence of this latter γδT cell subset was associated with complete responses observed in patients with B-cell precursor ALL after T-cell-depleted allogeneic HSCT [57,58]. Recently, Locatelli and colleagues carried out a single-center prospective trial investigating the outcome of children with acute leukemia transplanted from an HLA-haploidentical donor employing this new strategy of graft manipulation. Between September 2011 and September 2014, 80 pediatric patients aged below 21 years were enrolled in the trial. All children were given a fully myeloablative preparative regimen, mainly based on the use of total body irradiation (TBI). Remarkably, no patient received any pharmacological post-transplantation GvHD prophylaxis. Two children experienced primary graft failure. The cumulative incidence of skin-only, grade 1–2 acute GvHD was 30%, while no patient developed extensive chronic GvHD. Four patients died, leading to a cumulative incidence of TRM of 5%. Nineteen children experienced a recurrence of the original disease after the procedure, resulting in a 24% cumulative incidence of relapse. With a median follow-up of 46 months, the 5-year probability of the composite endpoint chronic GvHD-free, relapse-free survival (GRFS) is 71%. The authors compared the outcome of these 80 pediatric patients to those of 41 and 51 children given transplantation from an HLA-identical sibling or a 10/10 allelic-matched unrelated donor in the same center and period, demonstrating as haplo-HSCT after αβT- and B-cell depletion represents a competitive option for children with acute leukemia in need of urgent allograft [54]. In comparison with other studies mainly based on infusion of CD34^+^ cells, this paper did not document any favorable influence of NK cell alloreactivity and of donor KIR B-haplotype. It is likely that, using this approach, the NK-mediated GVL effect is partially concealed by other immune effector cells present in the graft, such as γδT cells [59]. Besides approaches of ex-vivo T-cell depletion, in the adult setting, the use of post-transplant cyclophosphamide (PT-Cy) as in-vivo GVHD prophylaxis contributed to the dramatic change in the use of haplo-HSCT, allowing safe infusion of unmanipulated T cell-replete grafts [60]. Indeed, as shown in several reports, PT-Cy selectively eliminates proliferating alloreactive T cells [61,62]. Whether and how the use of PT-Cy impacts on NK cells and their alloreactivity is still unknown. Very recently, Russo et al. characterized NK cell dynamics in PT-Cy haplo-HSCT recipients in two independent centers. After infusion of Cy, they documented a marked reduction of proliferating NK cells that were conversely present immediately after HSCT. This suggested a selective purging of dividing cells by PT-Cy. Remarkably, putatively alloreactive single KIR+ NK cells were also eliminated by PT-Cy. In this paper, the authors also evaluated the impact of donor NK cells alloreactivity in a series of 99 PT-Cy haplo-HSCT recipients. No significant difference in progression-free survival between patients with or without predicted NK alloreactivity (42% vs. 52% at 1 year, *p* = NS) was shown [63]. Overall, these results suggest a detrimental effect of PT-Cy on NK cells infused with the graft, jeopardizing the effect of their alloreactivity in this specific haploidentical transplantation setting.

While the antileukemic activity of NK cells and the role of KIR is well known and established by several groups, their impact in preventing graft failure and/or infections in patients affected by non-malignant disorders remain unclear. Bertaina et al. in 2014 conducted a pivotal study in 23 children with life-threatening non-malignant disorders receiving an αβT-cell depleted HLA-haploidentical HSCT, showing, with a median follow-up of 18 months, a 2-year probability of disease-free survival of 91.1% [64]. No patient developed visceral acute or chronic GvHD. In this cohort, mainly represented by primary immunodeficiencies, the cumulative incidence of TRM was 9.3%. Andreani and colleagues investigated if the absence of NK alloreactivity and/or a low B content value of donor KIR genotype may be correlated with graft failure in a cohort of 18 Thalassemia patients receiving haploidentical HSCT [65].To investigate if the presence of NK alloreactivity in the donor could improve patient outcome mediating an allorecognition of patient T lymphocytes and consequently limiting the cells mediating graft loss, they analyzed the donor KIR repertoire and donor/recipient HLA class I typing. A B content value of ≥2 was detected in 47% of the B/x donors. Their results showed no significant differences in the clinical outcome of the patients receiving the graft from a donor with NK alloreactivity or with a B content value ≥2.

## 4. Donor Specific Anti-HLA Antibodies: Dream or Reality?

“Natural” anti-HLA antibodies can be identified in healthy individuals, at a prevalence estimated to be between <1% and 5% [66]. Natural antibodies emerge from cross-reactions with common environmental antigens encountered by individuals all along their lives. They can be reactive against either denatured/cryptic HLA epitopes or native epitopes. The former interact with HLA molecules that are ill-configured because of natural instability or due to procedures used to produce, isolate, and adsorb the antigen on the beads [67]. Besides natural antibodies, patients may be alloimmunized by pregnancy, blood product transfusion, or previous transplantation. Moreover, in patients suffering from hematological diseases, anti-HLA immunization ranges from 19.6% to 39.4% [68]. Sensitization to donor alloantigens increases the risk of graft failure.

In the setting of HSCT, graft failure occurs more frequently in alternative donor transplantations, with an incidence that varies between 4% in MUD transplantations up to 20% in UCB and T cell-depleted haplo-HSCT [69]. Despite advances in HLA matching and supportive care, in view of the high treatment-related mortality associated with this event, graft failure remains a relevant problem. In the last few years, several papers have shown a link between donor-specific anti-HLA antibodies (DSAs) and graft failure in either mismatched related (haploidentical), matched and mismatched unrelated donor or UCB transplants, suggesting that anti-HLA sensitization should be routinely evaluated in HSCT with HLA mismatched donors. In one of these studies performed on 60 patients undergoing single-mismatch intra-familial or unrelated donor transplantation, the presence of DSAs detected by the serum cross-match technique was associated with a significantly higher risk of graft failure when the cross-match test was positive [70]. In a different study, the authors retrospectively reported 115 patients who had received HSCT from a donor with at least one mismatch among the A, B, C, DRB1, DQB1, or DPB1 loci after a myeloablative conditioning regimen (MAC) [71]. The results of this study showed that 24% of patients who did not engraft had pre-transplant DSAs positivity compared with 1% in patients who did engraft. More recently, several studies have been conducted in a T-cell replete haploidentical settings. Among 79 patients transplanted after a reduced intensity conditioning regimen (RIC), the authors reported a graft failure rate of 27% in the DSAs+ group (14%), compared with 3% rejection in the DSAs group [72]. Larger studies confirmed that the presence of DSAs was involved in the development of poor graft function (PGF). In a trial conducted on 345 patients prepared through MAC conditioning, 11.3% of patients showed positivity for DSAs. Multivariate analysis showed that the presence of DSAs was strongly associated with PGF (HR 10.575, 95% CI 2.029–55.117; *p* = 0.005) [73]. In a recent paper, Ciurea et al. determined prospectively the presence of anti-HLA Abs before transplantation in 592 MUD transplantation recipients using mixed screen beads in a solid-phase fluorescent assay. In this study, DSAs identification was performed using single Ag beads containing the cognate donor’s HLA-mismatched Ags. In this population, the proportion of graft rejection was statistically related to the presence of DSAs, while the presence of anti-HLA antibodies in the absence of DSAs did not predict graft failure. Moreover, the results of the abovementioned study indicate that the presence of DSAs in the patient’s pre-transplantation serum reactive with the donor HLA-DPB1-mismatched Ags is associated with increased risk for graft failure [74]. In a subsequent study, Ciurea et al. analyzed 122 haplo-HSCT recipients tested prospectively for DSAs presence. In addition, a retrospective analysis to detect C1q binding DSAs (C1q + DSAs) was performed on 22 allosensitized recipients. Out of 122 patients, 22 (18%) had DSAs, 19 of which were women (86%). Seven patients DSAs + (32%) rejected the graft while the incidence of graft failure was 4% in the DSAs-group (4%; *p* < 0.001). Median DSAs level at transplant for patients who failed to engraft was 10.055 mean fluorescence intensity (MFI) versus 2.065 MFI for those who engrafted (*p* = 0.007). Of 9 patients who were C1q positive in the initial samples, 5 patients remained C1q positive at time of transplant and experienced engraftment failure, whereas 4 patients became C1q negative pre-transplant and all engrafted the donor cells (*p* = 0.008) [75]. Overall these data suggest that complement mediated rejection plays a major role in antibody-mediated graft failure and that C1q should be tested routinely along with DSA levels prior to transplant. In addition, it appears that C1q negativity should be the goal of treatment, at least in C1q positive patients, and high DSA levels may not clear right away from circulation.

A similar experience was reported in children affected by hemoglobinopathies. Andreani M et al., in a subgroup of 18 thalassemia patients, were able to retrospectively evaluate by Luminex single antigen beads the presence of anti-HLA antibodies in the recipients, and in particular DSA. Anti-HLA antibodies were present in 6 of the 18 patients analyzed; 1 against class I antigens, 2 against class II and 3 against both, with a MFI varying from 1000 to 22,000. When they analyzed their specificity, they found that in three cases they could be classified according to donor HLA genotype as DSA with a percentage in their survey (17%) comparable to that reported in literature. Eight out of the 18 patients evaluated showed a secondary graft failure, while 10 reached a stable complete full engraftment. The analysis of correlation with graft rejection indicated that 5 of the 8 patients that lost their graft were positive for anti-HLA antibodies (62.5%), while only 1 out of 10 (10%) in the group of those with full donor engraftment revealed their presence, showing a statistically significant difference between the patients that rejected the graft and those showing a complete chimerism (*p* = 0.042). Among the patients in which they detected anti-HLA antibodies, 2 were DSA positive for class I and 1 was DSA positive for both class I and II. These 3 DSA positive patients belonged to the group of the 8 patients that lost the graft, while none of 10 patients with complete chimerism, were found positive for DSA (*p* = 0.068) [65].

Theoretically, recipient-specific anti-HLA antibodies also might affect the development of GvHD [22]. In a recent study, Delbos et al. evaluated 82 HLA class II mismatched unrelated transplant donor–recipient pairs [76]. In this cohort, 26 donors (32%) had at least one anti-HLA class II antibody in peripheral blood. The 2-year cumulative incidence of a first episode of either acute or chronic GvHD was significantly higher (88% versus 67%) in patients receiving a graft from an anti-class II immunized donor. This finding indicates that donor immunization against foreign HLA antigens could be a new criterion to consider for predicting the risk of GvHD after HLA-mismatched unrelated HSCT. Identifying the specificities of anti-class II antibodies revealed that 13 of 26 alloimmunized donors had recipient-specific antibodies, directed mainly against mismatched HLA-DPB1 alleles. Although intriguing, this observation needs to be confirmed in a larger cohort.

Although the mechanisms underlying the association between anti-HLA DSA and graft rejection in HSCT remain at least in part unclear, numerous studies report on a detrimental effect of DSA on HSCT engraftment without clearly identifying innocuous HLA loci. Screening for anti-HLA Abs is warranted when considering donors with mismatches, and attempts to reduce the Ab levels before transplantation deserve further investigation in hematopoietic stem cell transplantation (Table 1). Further trials, especially addressing the issues of a pre-transplant MFI threshold able to predict graft rejection, are needed. Moreover, since mismatches are most frequent in the DP locus even for 10/10 matched unrelated donors, the impact of anti-DP antibodies deserves to be deeply investigated.

## Figures and Tables

**Table 1:** Suggested criteria to consider for selecting the best hematopoietic stem cell transplantation donor.

Criteria to Consider (in Order of Importance)	Recommendation	Specific Suggestions
Evaluation of level matching for HLA-A, -B, -C, -DRB1, and -DQB1 loci	- Standard of care	- Consider the increased risk of grade II–IV aGvHD in pairs with antigen mismatch respect to those with allelic mismatch
HLA-C	- Consider the negative impact of a single HLA-C mismatches in unrelated HSCT	- Take into account the HLA-C compatibility, in view also of the HLA-B-C associations in specific haplotypes.
	- Detrimental effect of the presence of HLA-C mismatches at higher expression levels	- Consider that C*03:03/C*03:04 mismatched and the 8/8 matched groups have identical outcomes
		- If possible, evaluate the impact on aGvHD of the presence of HLA-C mismatches involving alleles with higher expression levels
HLA-DPB1	- Take into account the algorithm relative to permissive DPB1 mismatches	- Evaluate the permissive or non-permissive donor–recipient combinations of HLA-DPB1, in particular for what concern the risk of aGvHD and mortality
NK cells alloreactivity	- In case of haploidentical HSCT, evaluate NK cells alloreactivity based on the KIR-KIR ligand mismatch model	- Whenever possible, evaluate the alloreactive subset size (>5%).
KIR gene haplotypes	- Whenever possible, choose a B haplotype donor	- Whenever possible, choose a B haplotype donor with a B-content score > 2.
Donor specific anti-HLA antibodies	- Screening for anti-HLA Abs is warranted when considering donors with mismatches.	- Evaluate C1q binding DSAa and consider a pre-HSCT treatment for C1q+ patients.
		- Specific evaluation against mismatched HLA-DPB1 alleles are recommended.
